# Argus II retinal prosthesis for retinitis pigmentosa in the Middle East: *The 2015 Pan-American Association of Ophthalmology Gradle Lecture*

**DOI:** 10.1186/s40942-021-00324-6

**Published:** 2021-10-27

**Authors:** J. Fernando Arevalo, Saba Al Rashaed, Tariq A. Alhamad, Eman Al Kahtani, Hassan A. Al-Dhibi, for the KKESH Collaborative Retina Study Group, Marco Mura, Eman Al Kahtani, Sawsan Nowilaty, Saba Al Rashaed, Hassan A. Al-Dhibi, Yahya A. Al-Zahrani, Igor Kozak, Sulaiman Al-Sulaiman, Abdulelah Al-Abdullah, Ahmad Al-Bar, Yousef Al Dhafiri, Abdullah Al Qahtani, Khalid Al Rubaie, Saeed Al Shahrani, Maha Al Shehri, Badr Al Ahmadi, Abdulaziz Al Hadlaq, Majed Al Harbi, Abdulaziz Al Oreany, J. Fernando Arevalo

**Affiliations:** 1 Vitreoretinal Division, King Khaled Eye Specialist Hospital, P. O. Box 7191, Riyadh, 11462 Saudi Arabia; 2 Retina Division, Wilmer Eye Institute, Johns Hopkins University School of Medicine, Baltimore, MD USA

**Keywords:** Retinal prosthesis, Argus II, Retinitis pigmentosa, Functional vision, Blindness

## Abstract

**Background:**

To describe the outcomes of patients with retinitis pigmentosa (RP) who received the Argus II Retinal Prosthesis System.

**Methods:**

This retrospective, interventional case series evaluated 10 consecutive patients who received the Argus II retinal implant and underwent visual function tests with the system on and system off. The main outcome measures were safety (the number, seriousness, and relatedness of adverse events), and visual function measured by computer-based objective tests, including square localization (SL) and direction of motion (DOM). Secondary measures included functional vision performance, including orientation and mobility (O&M) tasks.

**Results:**

There were no intraoperative complications and all prostheses remained implanted at the end of follow up. The mean patient age was 41.3 years; mean duration of the implant in vivo was 2.1 years. One patient had a suture exposure over the coil suture tab and over the inferior case suture tab at 2 years postoperatively, which was managed successfully. One patient developed mild vitreous hemorrhage that resolved spontaneously. One patient developed high intraocular pressure postoperatively due to a tight scleral band (SB) that was managed successfully. Patients performed significantly better with the Argus II system on than off on all tasks.

**Conclusion:**

Patients who received the Argus II had a safety profile out to 4 years post-implantation that was markedly better than that observed in the pre-approval phase of the Argus II. In this population of RP patients, the Argus II retinal prosthesis provided useful visual function over several years that likely translates into improved quality of life.

*Trial Registration:* clinicaltrials.gov identifier, NCT00407602.

## Background

Retinitis pigmentosa (RP) is a group of disorders that are characterized by inherited, progressive loss of photoreceptor cells. The prevalence of RP has been reported as high as 1 in 372 in rural India to 1 in 4000 in western countries [1, 2]. Substantial data exist on the mutations and genes involved in RP patients from the Middle East in general and in the Kingdom of Saudi Arabia (KSA) in particular [3]. However, there are no epidemiologic studies reporting the prevalence of RP. The high rate of consanguineous marriages and significant number of papers published on mutation analysis in KSA suggest a relatively high prevalence of RP [4].

A significant number of clinical trials have investigated different approaches to manage this condition including gene therapy, stem cell transplantation and electronic neural prostheses in different locations in the eye [5–9]. The numerous management approaches indicate the lack of a definitive treatment for RP. However, the only treatment that is approved by the United States Food and Drug Administration (since February 2013) is the Argus II Retinal Prosthesis System (Second Sight Medical Products [SSMP], Inc., Sylmar, CA, USA). This prosthesis is a surgically implantable epiretinal device designed to provide artificial vision to patients with outer retinal degenerative disease with viable inner retinal cells and nerve fiber layer such as RP. Since June 2007, over 350 devices have been implanted at 25 centers in 12 countries worldwide. this number is unlikely to increase following SSMP’s announcement that the production of the Argus II Retinal Prosthesis System has been suspended in order to focus on optimizing the development of the Orion Cortical Prosthesis System (Second Sight Medical Products Inc., Sylmar, CA, USA).

The Argus II feasibility multicenter study (clinicaltrials.gov identifier, NCT00407602) in US and Europe enrolled thirty patients who underwent prosthesis implantation and were followed for 10 years postoperatively [10, 11]. A study on these 30 patients by da Cruz et al. reported the safety and performance of this study at 5 years post-implantation [12]. Patients performed significantly better with the Argus II system on than off on all visual function tests and functional vision tasks [12]. These results support the long-term safety profile and benefit of the Argus II system for patients who are blind due to RP. In Saudi Arabia, the Argus II system received limited approval [restricted to the King Khaled Eye Specialist Hospital (KKESH), Riyadh, Saudi Arabia] in June 2012 and full approval was received in June 2015.

The current study presents the functional and anatomic outcomes in 10 patients with RP that received the Argus II Retinal Prosthesis System at KKESH from 2013 to 2016.

## Material and methods

This retrospective consecutive interventional case series evaluated 10 patients (10 eyes) diagnosed with retinitis pigmentosa who underwent implantation of Argus II retinal prosthesis system from February 2013 to August 2016 at KKESH. Patients were referred from other centers within Saudi Arabia and some patients were self-referred. The KKESH Institutional Review Board (IRB)/Ethics Committee approval was obtained and patients signed an informed consent for this study. This study adhered to the tenets of the Declaration of Helsinki for research involving human subjects.

### The Argus II retinal prosthesis system

**Fig. 1:**
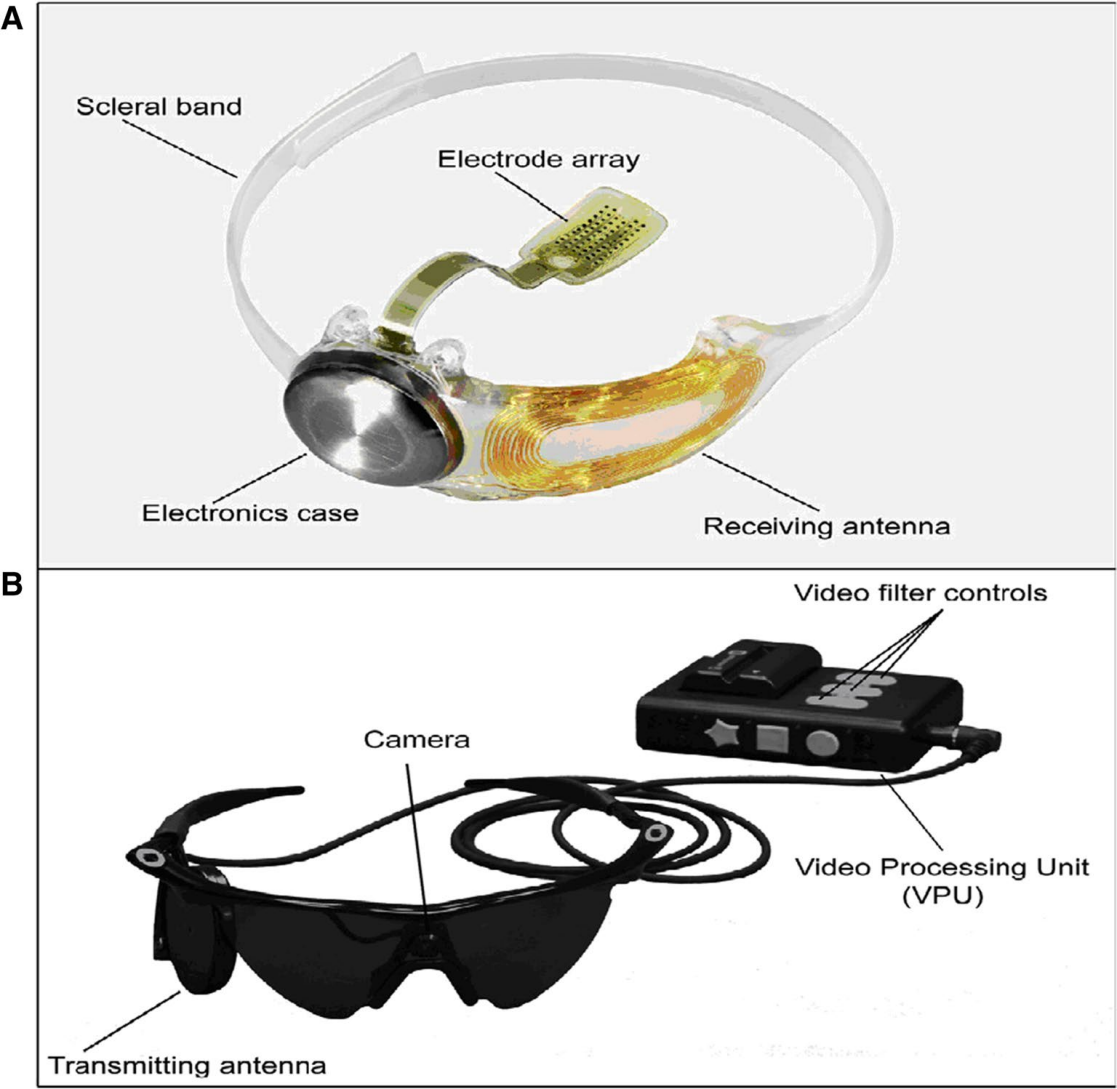
Photographs of the Argus II Retinal Prosthesis System (Second Sight Medical Products, Inc, Sylmar, CA). **A** the implanted components of the system and **B** the external (body-worn) components of the system

The external component of the Argus II consists of a glasses-mounted camera and a battery-powered video-processing unit that is worn on the patient’s body. The processing unit converts the camera-captured image into an electronic signal that is transmitted by cable to a transmitting coil located on the glasses. The implanted portion of the device (Fig. 1) consists of a receiving coil and an electronics capsule that wirelessly receives information from an external transmitting coil and is sutured to the sclera by an encircling scleral band. Data are sent via a small transscleral cable from the electronics capsule to the electrode array, which is firmly held to the retinal surface by a retinal tack. The array is a 6 × 10 grid of electrodes, with each electrode emitting electric pulses directly to the retinal surface. Direct retinal electric stimulation of the bipolar cells in the inner retina are then transmitted via the optic nerve to the visual cortex. This allows the patient to perceive spots of light. The maximum visual field obtained with the Argus II System is approximately 20°.

### Patient population

Inclusion criteria: patient age of at least 25 years, bare or no light perception [visual acuity worse than 2.9 logarithm of the minimum angle of resolution (logMAR)] in both eyes, a history of useful form vision, axial length between 21 and 26 mm, educated literate patients with at least high school education, a healthy, functioning optic nerve based on response to light.

Exclusion criteria: a physical condition that precluded prosthesis implantation, concurrent complicating ocular pathology, axial length less than 21 mm or longer than 26 mm, mental retardation or illiterate subjects.

Data were collected on patient demographics, ophthalmic examination, fundus photography, fluorescein angiography, optical coherence tomography (OCT) (Spectralis Heidelberg Retinal Angiography + OCT, Heidelberg Engineering, Heidelberg, Germany), and axial length measurement using ultrasonography. Data collected on preoperative and postoperative visual function tests included: the square localization (SL) test, direction of motion (DOM) test, grating visual acuity (GVA) test, and orientation and mobility (O&M) tasks. Intraoperative and postoperative complications were also recorded.

### Preoperative examinations and visual function tests

The SL test measures a patient’s ability to localize a white square on a black touch-screen monitor [10–12]. The size of the square (7.3 cm) and the contrast between the square and the computer screen (100%) do not vary, however, the location of the square on the computer screen changes. After positioning the patient 30.5 cm away from the screen, head scanning was used to localize the square on the screen. The subject was then asked to touch the middle of the white square and this process was repeated 40 times. The average difference between the center of the square and where the patient touched the screen (in cm), was automatically computed by the testing software. The DOM test measures a patient’s ability to detect motion. In this test, a white bar moves across a black computer screen [10–12]. The size (3.7 cm wide); contrast (100%); and speed (2000 ms screen crossing time) of the stimulus remains constant, but the direction of motion varies. The subject was asked to indicate the direction of motion on a touch-screen. Eighty trials were performed and the average difference between the stimulus angle and the response angle was automatically computed by the testing software.

### Postoperative examinations and testing

Follow-up visits were performed at 1 day, 1 week, and 1, 3, 6 and 12 months after surgery. At each follow-up visit, patients underwent a complete ophthalmic examination and a visual rehabilitation session was started at the 1-month postoperative visit. Optical coherence tomography (OCT) images were obtained at 1 month postoperatively to verify proper device positioning. Once the device was implanted, the video processing unit was individually calibrated for each patient. This was done using a special computer program that measured the perception threshold of each electrode and created a video configuration file. SL and DOM testing was performed at each postoperative visit beginning at 1 month postoperatively onwards with both eyes open and the device switched on. Grating visual acuity was tested only in the operative eye with the device switched on, patient mobility was tested beginning at 1 month postoperatively onwards with orientation and mobility (O&M) tasks [10–12]. The task consisted of asking the subject to locate a bright light on the corridor ceiling, find doors in a corridor, and to walk along a dark line (30 cm wide) on the floor or a white line on the pavement.

### Surgical technique for implantation of the Argus II retinal prosthesis system

Ten patients underwent surgery by 4 different surgeons (JFA, SAR, EAK, HAD). The eye with the worse visual acuity was selected for surgery. Prior to surgery, 8 mg of dexamethasone and 1000 mg of ceftriaxone were administered intravenously. Phakic patients underwent clear corneal phacoemulsification of the lens 1 month before prosthesis implantation or pars plana lensectomy during the Argus II surgery.

A 360° conjunctival peritomy was performed, and the 4 rectus muscles were isolated. The prosthesis system scleral band was passed under the 4 rectus muscles, the electronics package is located in the upper temporal quadrant, the coil in the lower temporal quadrant. The coil and the scleral band were fixed to the sclera by passing a 5–0 nylon suture (Alcon Laboratories, Fort Worth, TX, USA) through suture tabs located on the device in the temporal quadrants. In the nasal quadrants, the scleral band was fixed by 2 mattress sutures, and the scleral band was closed with a Watzke sleeve (FCI Ophthalmics Inc., San Francisco, CA, USA) in the upper nasal quadrant. A complete vitrectomy was performed using a 23-gauge valved entry system, and the posterior hyaloid was removed. Triamcinolone acetonide was used for better visualization of the vitreous. If present, epiretinal membranes were peeled. A further 5.2 mm wide sclerotomy parallel to the limbus was created in the superotemporal quadrant 3- 4.0 mm from the limbus, customized for each patient based on axial length. The sclerotomy was performed. The microelectrode array and cable were passed through the sclerotomy, and the nasal sclerotomy was widened to accommodate the 19-gauge tacking forceps. The array was positioned over the macular region with a silicone brush flute needle or end-gripping forceps. A customized tack was placed in a ring located on the array and, perpendicular to the globe wall, the array was tacked to the posterior pole superotemporal to the macula. Sclerotomies were closed with 7–0 vicryl (Ethicon, Somerville, New Jersey, USA), and a mattress suture was placed over the external aspect of the cable. The extraocular segment of the cable was covered with Tutopatch human pericardium or Tutopatch Bovine Pericardium (RTI Surgical Inc. Alachua, FL, USA). Tenon’s capsule and conjunctiva were sutured with 7–0 vicryl followed by intravitreal injection of vancomycin 1 mg/0.1 ml and ceftazidime 2.25 mg/0.1 ml. Cefazolin (100 mg) dexamethasone (2 mg) and lidocaine 4% (2 mL) were injected subconjunctivally. Patients were prescribed oral ciprofloxacin (500 mg) twice-a-day for 2 weeks, beginning 2 days before surgery. Postoperative topical medications included moxifloxacin (1 drop, 4 times a day) dexamethasone (1 drop, 4 times a day) and atropine 1% (1 drop, twice a day) for 2 weeks. Subjects took oral prednisolone (60 mg, once a day) for 2 weeks. Electrode impedance and waveform measurements were obtained prior to surgery, after scleral band positioning, after retinal-array tacking, and at the end of surgery. These tests were performed to ensure that each of the 60 electrodes was functioning properly before, during and after prosthesis implantation.

The primary outcome measures were safety (the number, severity, and whether adverse events were device-related) and visual function, as measured by 2 computer-based, objective tests, SL and DOM. Secondary measures included functional vision performance, including orientation and mobility (O&M) tasks.

## Results

**Fig. 2:**
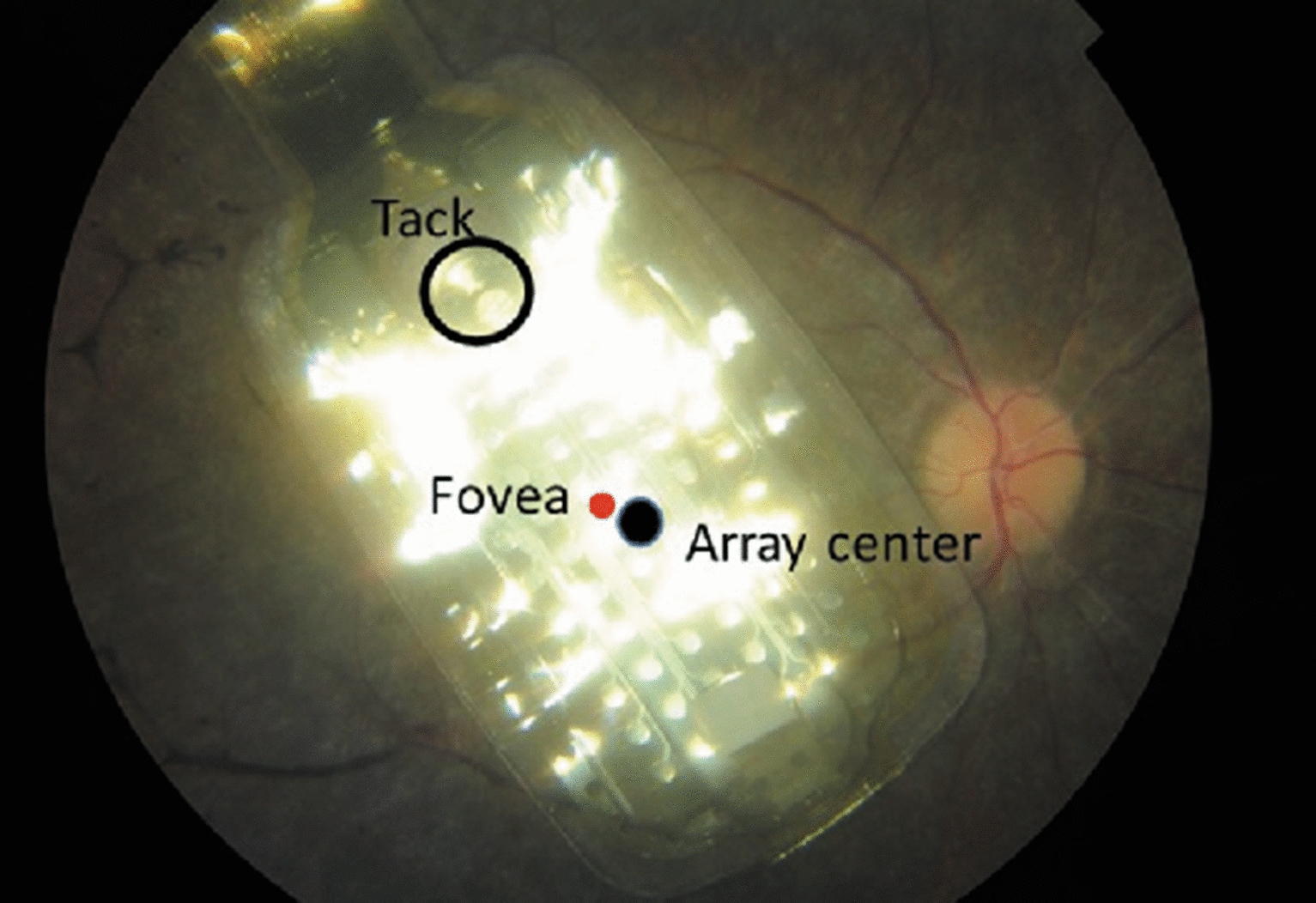
Fundus photo of the prosthesis array and approximate foveal location (red dot). The electrode array is centered between the superior and inferior arcades. The distance from the fovea to the center of the array was 0.8 mm

The implantation surgery was uneventful in all cases. At the end of follow up, all 10 patients retained the prosthesis. Seven patients were male and the mean age of the study sample was 41.3 years (range, 26.0–55.0 years). The prosthesis was implanted in 6 right eyes and 4 left eyes (Fig. 2; Table 1). Mean duration of follow-up was 2.1 years, ranging from 4 months to 3.8 years. The visual acuity of all patients was no better than light perception. Preoperative monocular visual acuity was worse than 2.9 logMAR in all patients. The mean axial length of the operative eyes was 23.96 mm (range, 22.76 mm to 25.83 mm). Six eyes were pseudophakic, 3 phakic eyes underwent pars plana lensectomy during prosthesis implantation surgery, and 1 eye had previous surgical aphakia.

**Table 1:** Clinical data of the 10 patents implanted who received the Argus II system

Patient no	Eye	Gender	Age (years)	Axial length (mm)	Lens Status	Peeling of posterior hyaloid	Postoperative complications	No. of functioning electrodes	No. of rehab sessions
1	Right	Male	45	24.96	Pseudophakic	YES	Conjunctival erosion	57	8
2	Left	Female	52	24.70	Aphakic	NO	Electrical Array Malposition	47 +	7
3	Right	Female	29	22.76	Pseudophakic	NO	NO	59	6
4	Left	Male	45	25.83	Pseudophakic	YES	NO	56	3
5	Right	Male	38	23.75	Pseudophakic	NO	NO	37*	3
6	Left	Male	55	24.05	Pseudophakic	YES	Mild vitreous hemorrhage	55†	2
7	Right	Male	33	24.20	Phakic	YES	NO	58	5
8	Left	Male	47	22.76	Phakic	YES	High IOP	60	2
9	Right	Female	26	23.74	Pseudophakic	NO	NO	60	0
10	Right	Male	43	22.90	Phakic	NO	NO	59	0

+ loss of 10 electrodes channels during electronic array tacking

*18 electrodes with high impedance value were observed at the end of the surgery. Electrode channels were damaged during the extra ocular placement of the device

^†^After the tack insertion into the vitreous chamber, the surgeon observed that the tack was misaligned with the tack tool tip. When the surgeon removed it from the vitreous chamber, the tack dislodged from the tack tool and fell inside the vitreous cavity. The tack was managed as a foreign body and removal procedure was performed

*IOP* Intraocular pressure, *No.* Number, *mm* millimeters, *RE* Right eye, *LE* Left eye, *Gen* Gender

Mean surgical time was 2 h and 52 min (range, 2–4 h 7 min). Six patients had tight adherent posterior hyaloid and/or epiretinal membrane that required peeling. One patient had a preplaced scleral band and subretinal band at the macula and we had difficulties tacking the electronic array due to irregularity of the retinal surface. Electrode channel damage occurred intraoperatively in 3 patients; 2 patients had difficulty during retinal tacking (cases 2 and 6) and in 1 patient (case 1) it was possibly related to exposure of the implant to a diathermy current intraoperatively.

### Anatomical outcome and postoperative complications

**Fig. 3:**
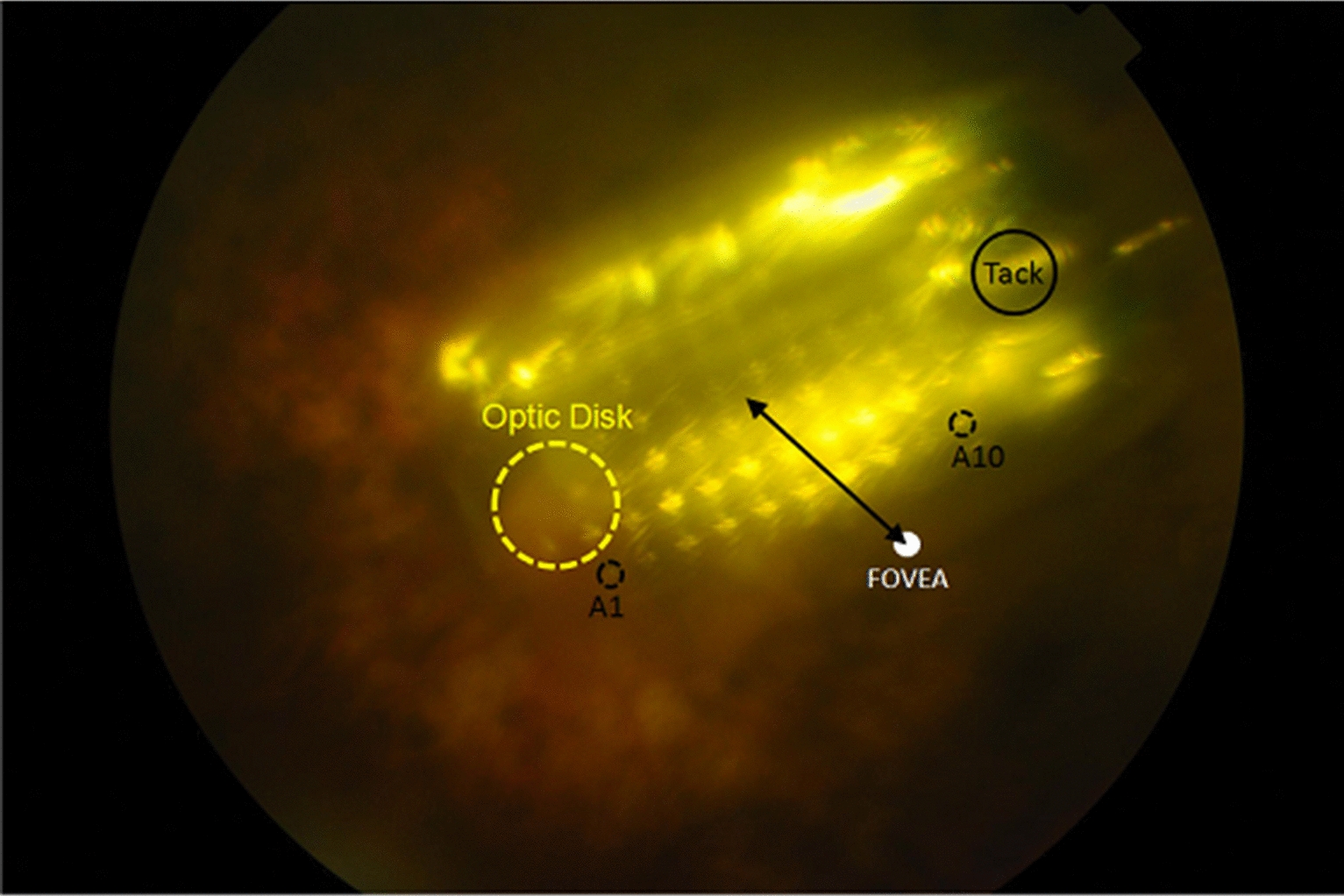
Fundus photo of the prosthesis array and approximate foveal location. The electrode array is superior to the fovea and slightly nasal to the optic disk. The distance from the fovea to the center of the array was 2.8 mm

**Fig. 4:**
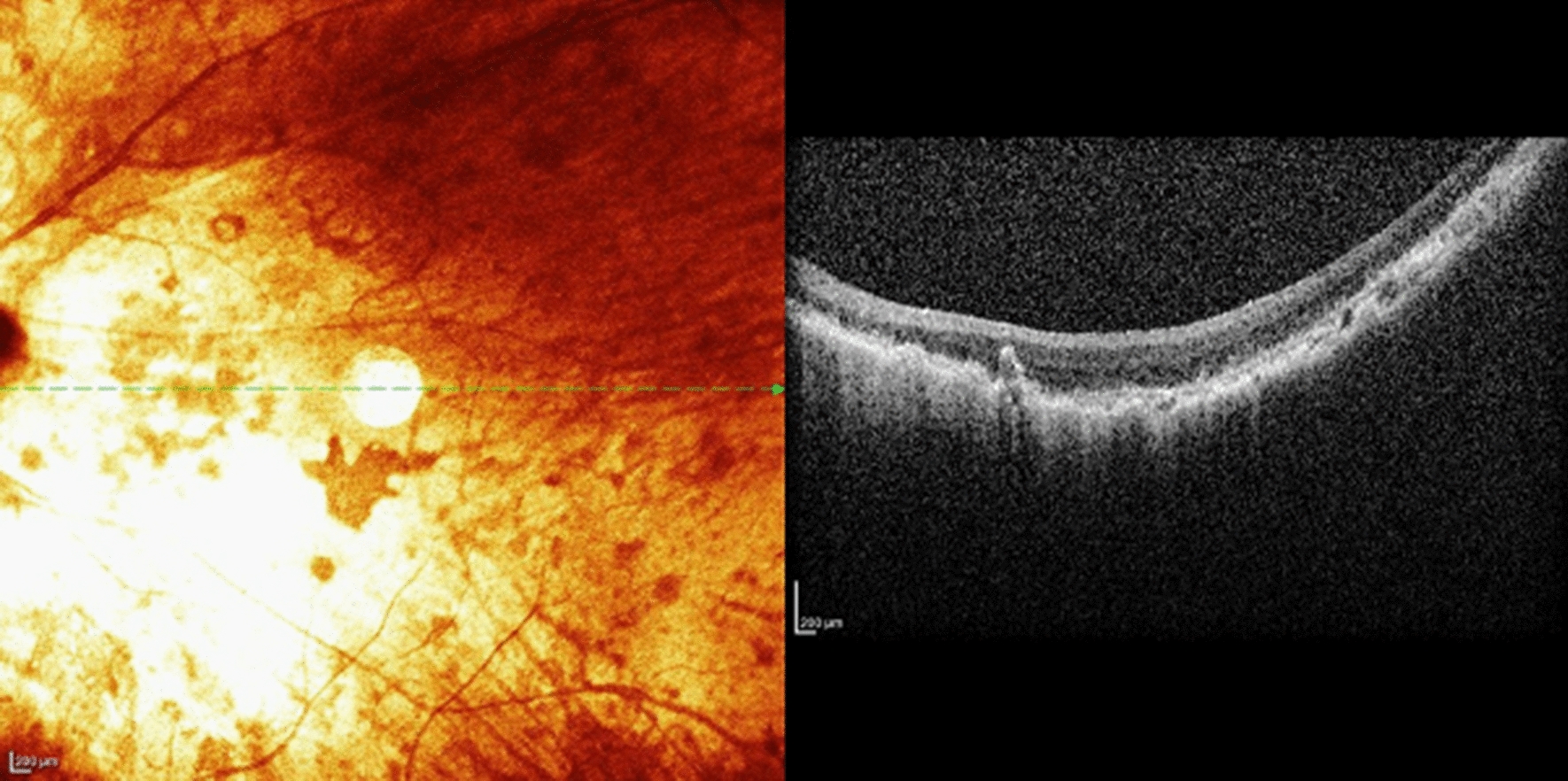
Case 2 optical coherence tomography (OCT) did not show a subretinal band and retinal fold

**Fig. 5:**
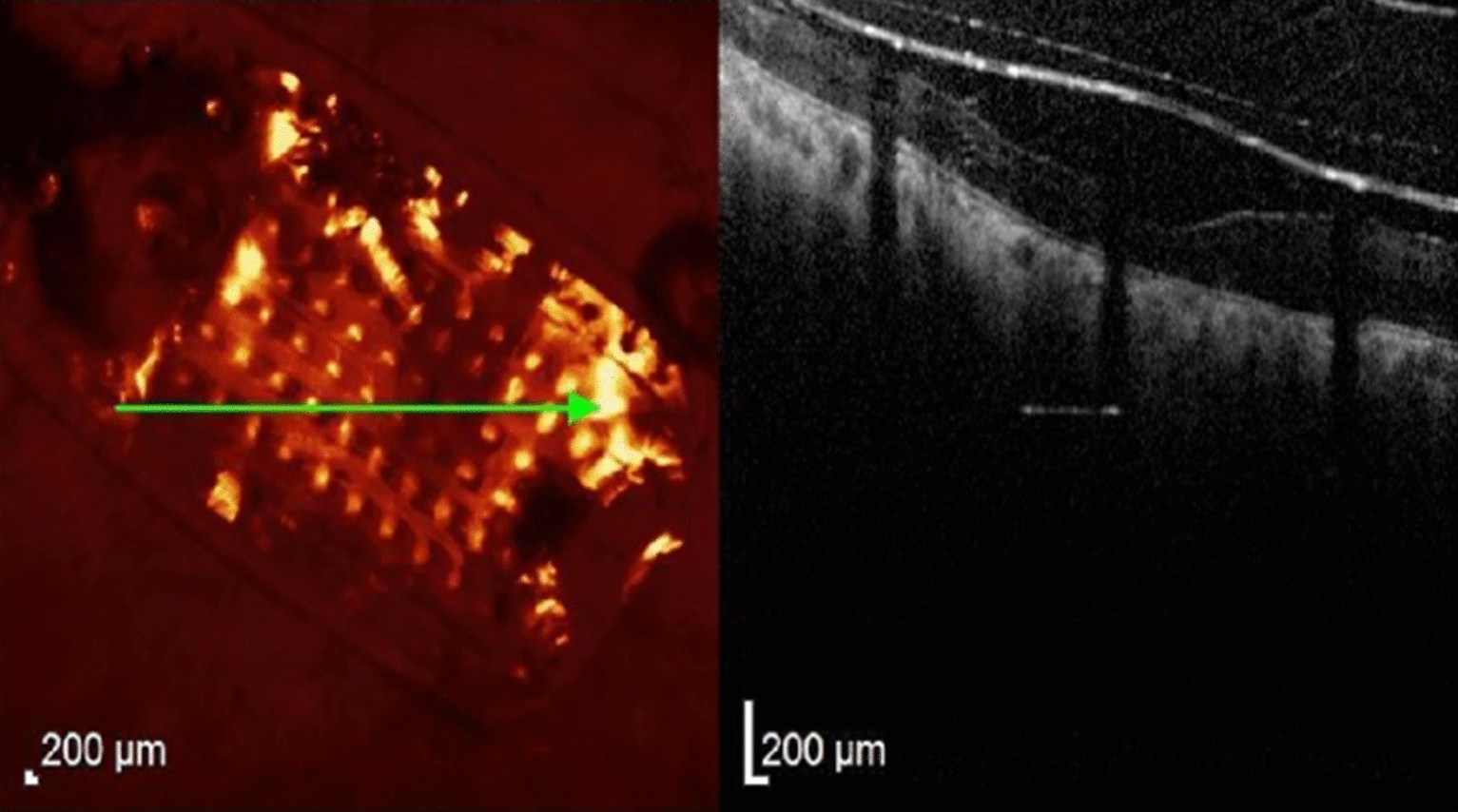
Optical coherence tomography (OCT) demonstrating perfect apposition of the implant to the retina

During the 4 years follow-up period none of the patients had any serious adverse events that required further surgery or required device explantation. There were no cases of retinal detachment or endophthalmitis. The implant was mispositioned in one patient (Case 2) due to a macular fold (Figs. 3 and 4). Postoperatively, this patient experienced minor improvement in visual function. One patient (case 6) developed a mild vitreous hemorrhage postoperatively, likely secondary to intraoperative manipulation caused by a dislodged tack. This complication resolved spontaneously after 2 weeks. One patient experienced suture exposure over the coil suture tab and over the inferior case suture tab at 2 years postoperatively. This was managed by conjunctival suturing and pericardium patch graft placement. One patient experienced high intraocular pressure postoperatively due to a tight scleral band which was remedied by relaxation of the scleral band. The intraocular pressure returned to normal limits without anti-glaucoma medications. Postoperative OCT imaging showed that the array was well positioned in all patients (Fig. 5).

### Visual function and performance outcomes

**Fig. 6:**
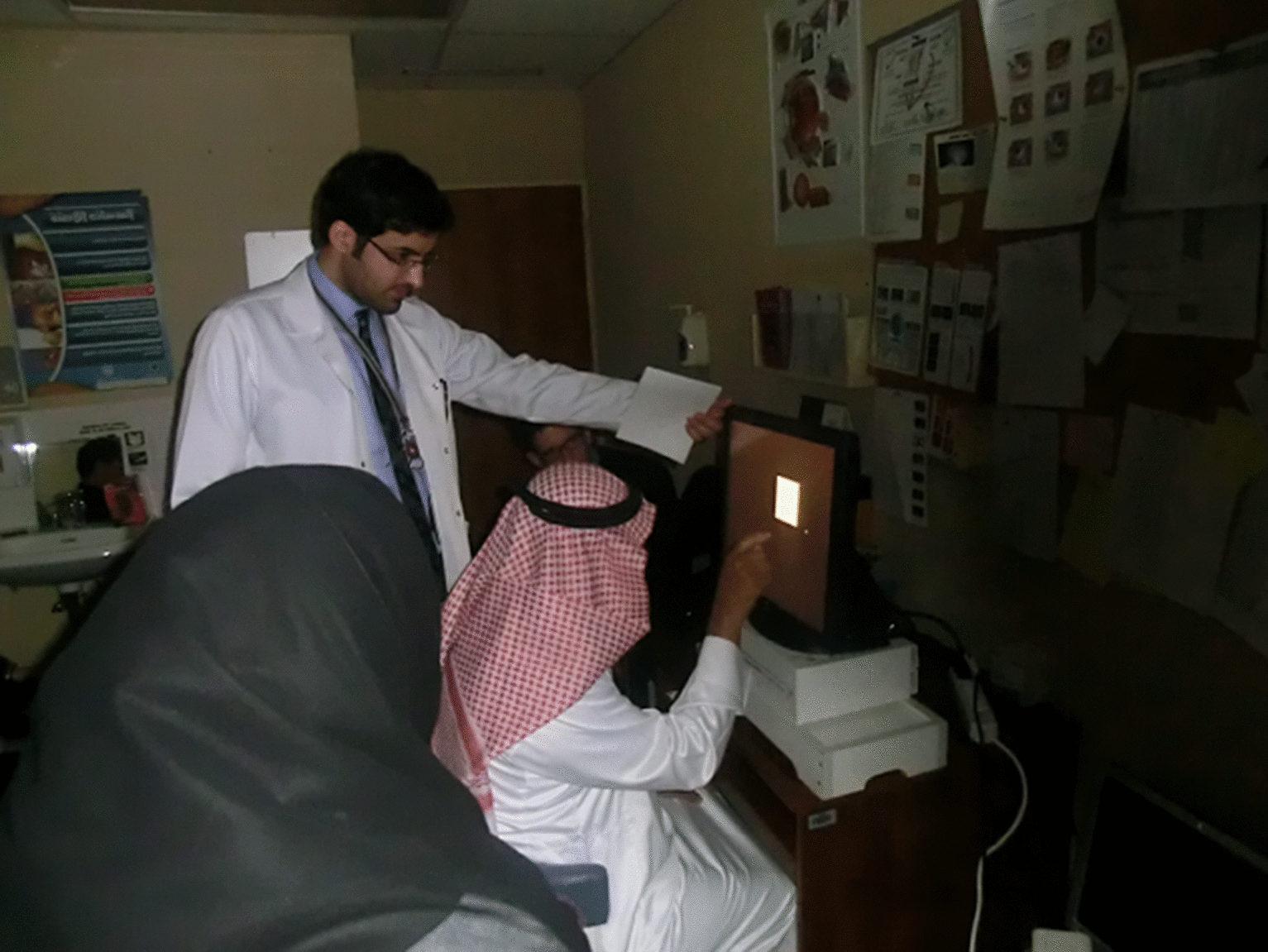
The square localization (SL) test, which measures a patient’s ability to localize a white square on a black touch-screen monitor

**Fig. 7:**
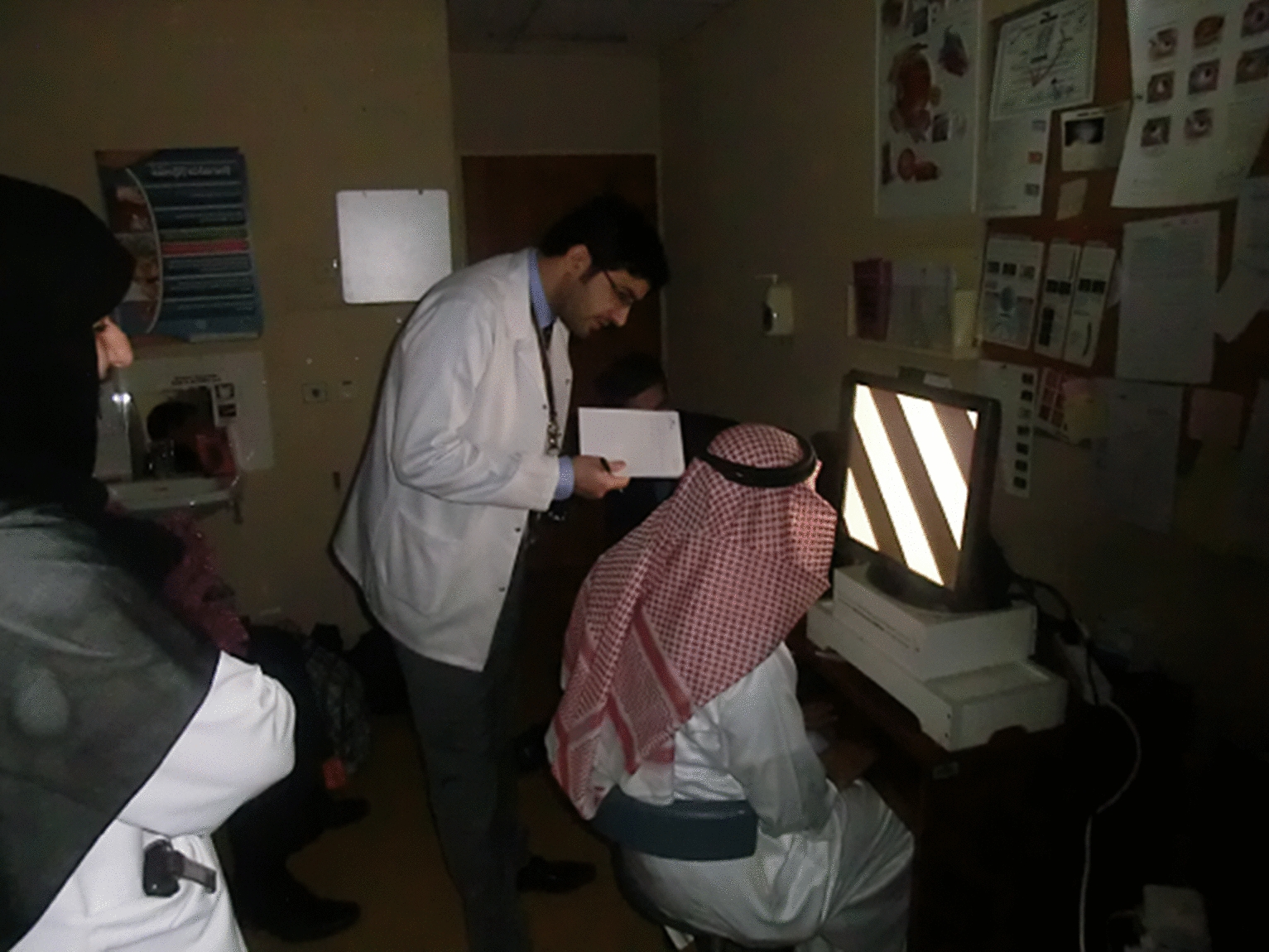
A direction-of-motion (DOM) test, which measures a patient’s ability to detect motion

Eight out of 10 patients performed significantly better with the Argus II system on than off on visual function tests and functional vision tasks. The mean error (± SD) for SL was 8. 83 ± 0.9 cm with the system on and 16.11 ± 1.5 cm with the system off (Fig. 6) and this was statistically significant (p < 0.001). The mean error (± SD) for DOM across was 81.32 ± 6.2° with the system on and 90.60 ± 5.9° with the system off (*P* > 0.05) (Table 2 and Fig. 7). Eight of 10 (80%) patients achieved visual function improvement as demonstrated by the SL results and 4 of 10 (40%) patients experienced a large improvement in visual function. O&M outcomes were obtained in the 10 patients (Table 3). These patients were able to locate a bright light on the ceiling, avoid/detect obstacles, and all of these patients could detect a person in front of them.

**Table 2:** Visual function results

Patient no	Duration of implantation (months)	Pre-Op visual acuity	Post-Op visual function test (number of correct responses, out of 40 for SL and 80 trials for DOM)			Post-Op visual acuity	Comment
			SL	DOM	GVA		
1	47.0	BLP	24/40	46/80	2.9 LogMAR	CF	Large improvement
2	47.0	BLP	17/40	9/80	3.0 LogMAR	LP	Improvement
3	38.0	LP	23/40	15/80	2.9 LogMAR	HM	Large improvement
4	38.0	BLP	11/40	37/80	2.9 LogMAR	CF	Large improvement
5	38.0	BLP	11/40	15/80	3.0 LogMAR	LP	Improvement
6	25.0	BLP	NA	NA	NA	BLP	Stable
7	25.0	LP	36/40	21/80	3.0 LogMAR	LP +	Improvement
8	9.0	LP	21/40	34/80	2.9 LogMAR	CF	Large improvement
9	5.0	LP	7/40	21/80	3.0 LogMAR	LP	Stable
10	5.0	LP	31/40	27/80	3.0 LogMAR	LP +	Improvement

Performance levels are the best out of 4 visits

*No.* Number, *Pre-op* Preoperative, *Post-op* Postoperative, *BLP* Bare light perception, *LP* Light perception, *LP +*  = Light perception and projection, *HM* Hand movements, *CF* Counting fingers, *SL* Square localization, *DOM* Direction of movement; *GVA* Grating of visual acuity, *LogMAR* logarithm of the minimum angle of resolution, *NA* Not available

**Table 3:** Rehabilitation outcomes with the system on

Patient No	Implant Duration (months)	Implanted Eye	Person detection in front of patient	Tracking/counting people in front of patient	Avoid/detect obstacles in front of patient	Walking by following a line and/or lights
1	47.0	OD	+	+	+	+
2	47.0	OS	+	+	+	+
3	38.0	OD	+	+	+	+
4	38.0	OS	+	+	+	+
5	38.0	OD	+	+	+	+
6	25.0	OS	+	NA	+	+
7	25.0	OD	+	+	+	+
8	**9**	OS	+	+	+	+
9	**5**	OD	+	+	+	+
10	**5**	OD	+	+	NA	NA

*No.* Number, *NA* Data not available,  + Able to achieve

Patients were not able to perform any of these tasks with the system off

## Discussion

In this study, the implantation of the Argus II retinal prosthesis was safely performed in all patients. To date, none of patients have required an explantation of the prosthesis. The most common adverse events after Argus II implantation include conjunctival erosion/dehiscence, hypotony, and endophthalmitis [13]. In our series, only one patient developed conjunctival erosion and was managed by suturing of the conjunctiva. A tight scleral band (SB) caused high intraocular pressure in one patient. The intraocular pressure returned to normal limits after relaxation of the SB. This complication has not been previously reported after Argus II implantation hence we recommend assessment of the SB tension before the end of surgery. One patient had mild vitreous hemorrhage that resolved spontaneously without intervention.

Electrode channel damage occurred intraoperatively in 3 patients, two patients had difficulty during retinal tacking; one patient (case 2) had an irregular macular surface caused by a subretinal band and an adjacent fold that was not recognized before surgery. This patient had postoperative malposition of the electrical array. We elected to observe this patient and there was some improvement of visual function and the O&M tests were improved. Another patient (case 6) had a dislodged retinal tack in the vitreous that was removed and a new tack was placed. This patient was lost to follow up but returned at 4 months postoperatively, and his O&M performance showed some improvement. In the third patient (case 1) electrode channel damage occurred due to exposure to diathermy current that was used to cauterize episcleral bleeding. Hence, we do not recommend diathermy during the implant procedure. However, this patient had a large gain in visual function tests, postoperatively.

Patients performed significantly better with the Argus II system on than with the system off on all visual function tests and functional vision tasks, and in 80% of cases, the visual function improved. Additionally, all patients were able to use the device for daily-life conditions and to locate a bright light on the ceiling, detect obstacles, and in most cases, even detect a person in front of them. These results concur with previous studies Ahuja et al. and Dorn et al. report that touching the square or tracing within 15° from the true DOM is considered correct [11, 13–15]. Hence, a binomial distribution can determine whether the subjects performed above chance. For example, given the size of the square and the screen puts the likelihood of touching the square by chance at (7.3/28.7) x (7.3/38.3) = 0.0485, and the cut-off for an above-chance performance at 5 out of 40; touching the square 5 or more times in 40 trials by chance has a probability of 0.0453. For the DOM test, the likelihood of a correct response by chance is (2 × 15/360) = 0.0833, the required number of correct responses is 12—tracing within 15° of the true direction 12 or more times out of 80 has a probability of 0.033. In other words, all subjects except #6 performed above chance on the SL test, and all except #2 and #6 on the DOM test.

Patient selection, counseling for realistic patient expectations, and preoperative retinal assessment are crucial steps for a successful outcome with Argus II implantation [16]. Case 2 in our series highlights these points. This patient had a small pupil with posterior synechia, which may have precluded an adequate preoperative assessment. Our experience also indicates that initially, some of the patients did not adhere to the recommended follow up for visual rehabilitation sessions. Patients gave several reasons related to lengthy commute to the hospital, motivation, and high expectations. This issue was addressed by inviting all the patients simultaneously to the hospital and allowing direct interaction with the surgeons, Argus II experts, and rehabilitation specialists. After this meeting we noticed a dramatic increase in patient compliance to follow up and all the patients seemed increasingly motivated to use the devise daily.

There are some limitations to this study. The small numbers and retrospective nature of this study precluded in depth statistical analysis because additional data were unavailable. Hence the statistical power of this study remains limited, and did not allow for a statistical difference in all visual function tests. A larger number of patients would need to be studied with longer follow up. In addition, we did not include a quality of life assessment questionnaire. In future prospective clinical trials of artificial vision, a quality of life assessment questionnaire before and after the procedure would be desirable. However, our patients were satisfied with the results and felt that the Argus II helped them to perform their visual tasks better and this is the first time this is has been demonstrated in a Middle Eastern population.

## Conclusions

In conclusion, our results demonstrate the efficacy and safety of the Argus II epiretinal prosthesis system, which can be an option to restore some vision in blind eyes with retinitis pigmentosa. Strict patient selection criteria are highly recommended for excellent patient outcomes.

## Data Availability

The datasets generated and/or analyzed are available from the corresponding author on reasonable request.

## Appendix

The following investigators belong to The King Khaled Eye Specialist Hospital (KKESH) Collaborative Retina Study Group:

*The King Khaled Eye Specialist Hospital (KKESH)*: Marco Mura, MD (PI), Eman Al Kahtani, MD, Sawsan Nowilaty, MD, Saba Al Rashaed, MD, Hassan A. Al-Dhibi, MD, Yahya A Al-Zahrani, MD, Igor Kozak, MD, Sulaiman Al-Sulaiman, MD, Abdulelah Al-Abdullah, MD, Ahmad Al-Bar, MD, Yousef Al Dhafiri, MD, Abdullah Al Qahtani, MD, Khalid Al Rubaie, MD, Saeed Al Shahrani, MD, Maha Al Shehri, MD, Badr Al Ahmadi, MD, Abdulaziz Al Hadlaq, MD, Majed Al Harbi, MD, Abdulaziz Al Oreany, MD.

*Wilmer Eye Institute, Retina Division, Johns Hopkins University, Baltimore, MD, USA*: J. Fernando Arevalo, MD, PhD, FACS (PI).

## Abbreviations

SL: Square localization
DOM: Direction of motion
O&M: Orientation and mobility tasks
RP: Retinitis pigmentosa
SB: Scleral band
KSA: Kingdom of Saudi Arabia
SSMP: Second Sight Medical Products
KKESH: King Khaled Eye Specialist Hospital
IRB: Institutional Review Board
logMAR: Logarithm of the minimum angle of resolution
OCT: Optical coherence tomography
GVA: Grating visual acuity
